# 4-Chloro-2-Methyl-Phenoxy Acetic Acid-Induced Liver Injury: A Case Report

**DOI:** 10.7759/cureus.38363

**Published:** 2023-04-30

**Authors:** Izzathunnisa Rahmathullah, KT Sundaresan, Kumara GMSS

**Affiliations:** 1 General Medicine, Teaching Hospital Batticaloa, Batticaloa, LKA; 2 Clinical Sciences, Eastern University of Sri Lanka, Batticaloa, LKA

**Keywords:** r-value, cholestasis, herbicide, drug-induced liver injury, 4-chloro-2-methyl-phenoxy acetic acid

## Abstract

Chlorophenoxy herbicides are a major herbicide used by Sri Lankan farmers. One common variety of chlorophenoxy herbicides is 4-chloro-2-methyl-phenoxy acetic acid (MCPA). Reports of clinical toxicity of MCPA in humans are limited. To our knowledge, liver damage is a rare manifestation of MCPA poisoning, which has not yet been reported in Sri Lanka. We report a case of a farmer who presented with a heterogeneous pattern of liver damage after being exposed to the MCPA pesticide for six months and who later fully recovered with treatment.

## Introduction

Herbicide poisoning is still a significant issue in Sri Lanka, especially among agricultural communities. One of the most common chlorophenoxy herbicides used by farmers is 4-chloro-2-methyl-phenoxy acetic acid (MCPA) [1]. Some of the unusual presentations that have already been documented in the literature include acute lethal renal toxicity, respiratory arrest, gastrointestinal symptoms, and neuromuscular weakness [2]. Numerous pathophysiological theories for direct toxicity have been put forth, despite the fact that the underlying mechanism of organ damage is unknown [2].

In our case, a farmer who had been exposed to the herbicide MCPA for six months was admitted to the hospital with evidence of obstructive liver injury. The farmer was eventually found to have a mixed pattern of liver abnormalities and histological evidence of toxin-mediated liver injury.

## Case presentation

A 33-year-old previously healthy man from Ampara District of Sri Lanka presented with yellowish discoloration of his eyes for two weeks. The yellow discoloration progressed rapidly and was associated with nausea, vomiting, epigastric pain, and generalized itchiness of the body. The urine was dark, but his bowel opening was normal in color and consistency. There were no respiratory, neurological, or constitutional symptoms or weight loss. No features suggestive of autoimmune diseases, such as joint pain, photosensitivity rashes, oral ulcers, loss of hair, dry eyes, or dry mouth were present.

There was no history of outside food consumption, blood transfusions, high-risk sexual behaviors, illicit drug abuse, over-the-counter medications, or Ayurvedic drug treatments. There was no history of alcohol consumption or smoking. The hygienic measures in food preparations and drinking water were acceptable and adequate. Previous instances of eye darkening and inherited liver or neurological problems did not exist.

He worked as a farmer for the last six months after completing his education. He had routinely sprayed MCPA herbicide on his paddy for the past six months, and one month before admission, he was unexpectedly exposed to a considerable dose of that particular herbicide while he was mixing up the chemical as a lot of mist blew out and sprayed onto his body, including his face.

**Figure 1:**
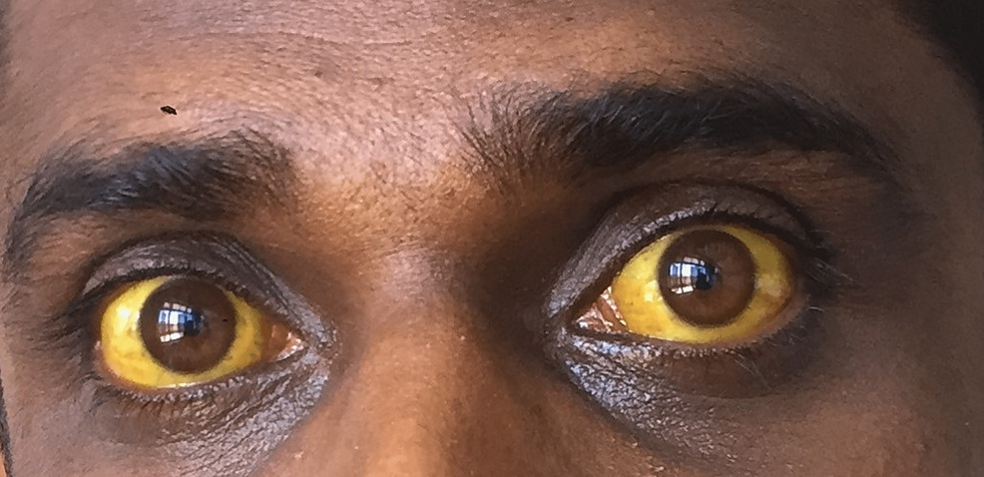
Deep icterus on admission

On admission, the patient was conscious and rational without any features of hepatic encephalopathy. The body temperature was normal. The sclera was not pale but was found to have deep icterus (Figure 1).

No Kesher-Fleischer rings, xanthomata, or xanthelasma was present. The fundus was normal. There were no rashes, clubbing, or lymphadenopathy. His BMI was within the normal range.

No peripheral stigmata of chronic liver cell disease or chronic alcohol use was noted. However, a nontender firm hepatomegaly measuring about 5 cm from the right lower costal margin was detected. No splenomegaly or free fluid was identified. With a blood pressure of 120/70 mmHg, a pulse rate of 80 beats per minute, and a saturation of 100% on room air, the patient’s hemodynamics were stable. The rest of the respiratory, circulatory, and neurological systems were normal. All the basic blood investigations including full blood count, inflammatory markers, and renal functions were normal in range.

Hepatitis A, B, and C and his HIV viral test results were negative. No cytomegalovirus and Epstein-Barr virus antibodies were found. An extensive autoimmune screening assessed anti-nuclear, anti-mitochondrial, anti-smooth muscle, and anti-liver-kidney-muscle antibodies and found them to be normal. No vasculitic markers, such as perinuclear (P) and cytoplasmic (C) antineutrophilic cytoplasmic antibody (ANCA), were found. His serum ceruloplasmin level was normal with the range of 20 mg/dl.

**Figure 2:**
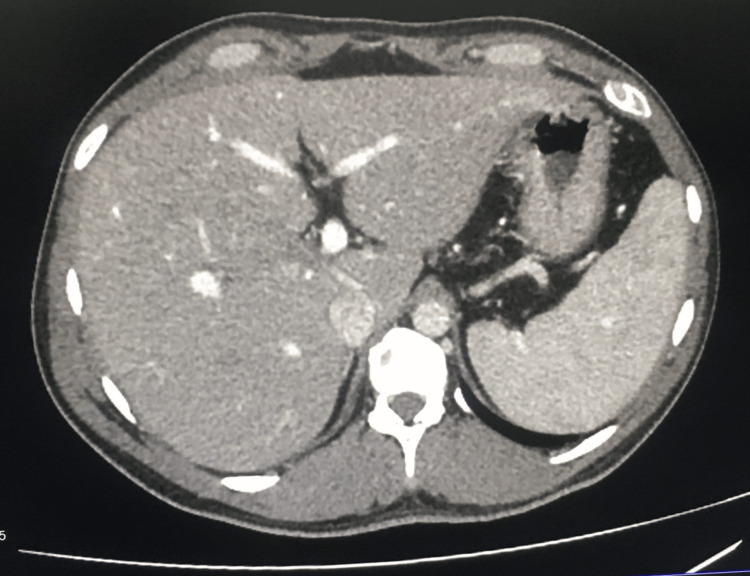
Contrast CT abdomen with mild hepatomegaly and early fatty liver changes

The CA 19.9 and alpha-fetoprotein were both normal. The serum immunoglobin G level was 1006 mg/dl, which was normal. The 2D echocardiogram and chest X-ray were normal. In the ultrasound scan of the abdomen, mild hepatomegaly was noted, and the spleen was normal. The intra- and extrahepatic bile ducts and the common bile duct were normal without any features of obstruction or dilatation. No stones or sludge were detected. His contrast-enhanced computed tomography (CECT) of the abdomen revealed grade 1 fatty liver alterations and mild hepatomegaly. No obstructions or localized lesions were found (Figure 2).

The liver function test revealed a mixed pattern of liver injury. However, with time and treatment, it started to improve gradually (Table 1).

**Table 1:** Full liver function test values since admission to discharge INR: International normalized ratio; ALT: Alanine transaminase; ALP: Alkaline phosphatase; ULN: Upper limits of normal; GT: Glutamyl transferase.

Biochemical parameters	Reference value	Patient's value on admission		Day 3	Day 7	Day 14	Day 20	Day 25	Day 30
Alanine transaminase (U/liter)	(12–78)	560		452	390	304	240	186	103
Aspartate transferase (U/liter)	(15–37)	304		179	154	140	114	103	60
Alkaline phosphatase (U/liter)	(46–116)	218		188	140	114	110	90	75
Total bilirubin (µmol/liter)	(3.4–17.1)	511		418.6	440	249	114	109	57
Direct bilirubin (µmol/liter)	(0–3.4)	306		240.6	297	194	89	67	38
Total protein (g/liter)	(64–82)	76		60	65	62	55	51	42
Albumin (g/liter)	(34–50)	28		25	30	40	40	36	28
INR	(1.1)	1.9		1.66	1.60	1.32	1.2	1.04	1.04
Gamma GT (U/liter)	(15–85)	172		280	142	138	118	104	77
R-value (ALT/ULN) (ALP/ULN)	(2:cholestatic, 2-5: mixed, >5: hepatocellular)		3.8	3.6	4.1	4.0	3.2	3.1	2.0

**Figure 3:**
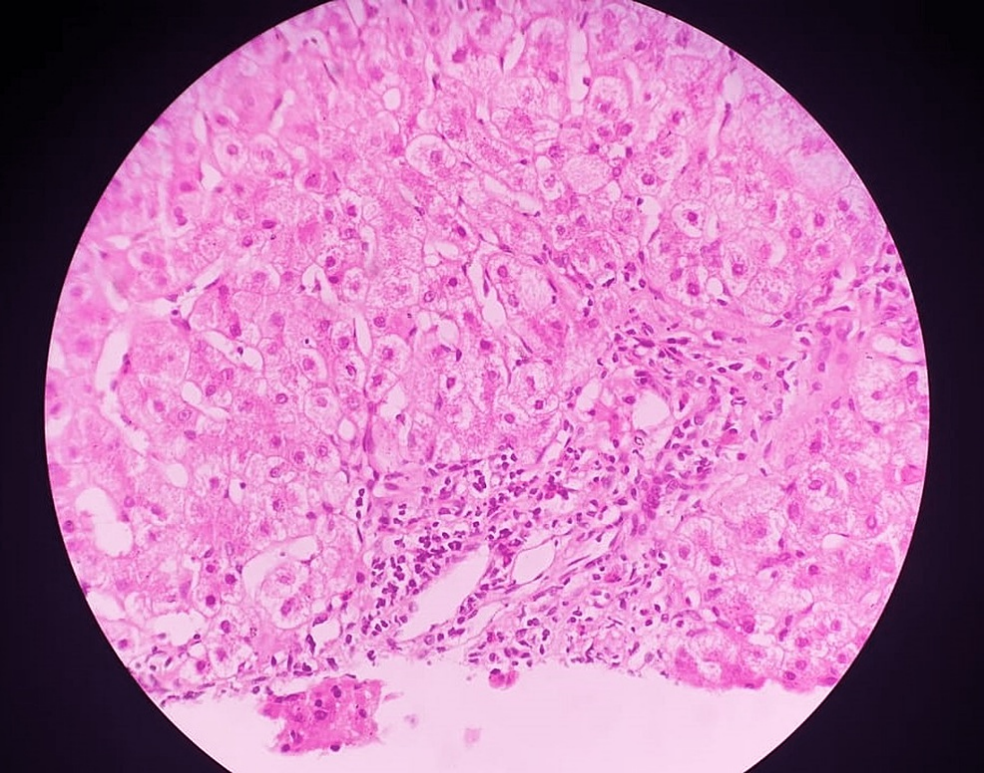
Drugs/toxins-induced hepatotoxicity indicated by a moderate portal inflammatory infiltrate consisting of neutrophils and eosinophils. Slides are stained with hematoxylin and eosin. Bar = 100 µm.

An ultrasound scan-guided liver biopsy was done, which showed moderate portal inflammatory infiltrates of lymphocytes, polymorphic nuclear cells, and many eosinophils with mild cholestasis and hepatic parenchymal focal necrotic inflammation consistent with drug or toxin-related hepatotoxicity (Figure 3).

The history suggested continuous exposure to herbicide for a significant period, so MCPA-induced liver injury was highly suspected. Apart from the elimination of the toxin, as his bilirubin was continuously high, he was started on pulse doses of 500 mg IV methylprednisolone daily for three days followed by oral prednisolone of 40 mg daily. In addition, 250 mg of ursodeoxycholic acid was administered daily to treat the ongoing itching.

The patient responded to the medications gradually. Three weeks after admission, his liver function started to improve, and we were able to discharge him even though the bilirubin level was still on the higher side. On discharge, we clearly instructed the patient to completely avoid being exposed to the herbicide.

**Figure 4:**
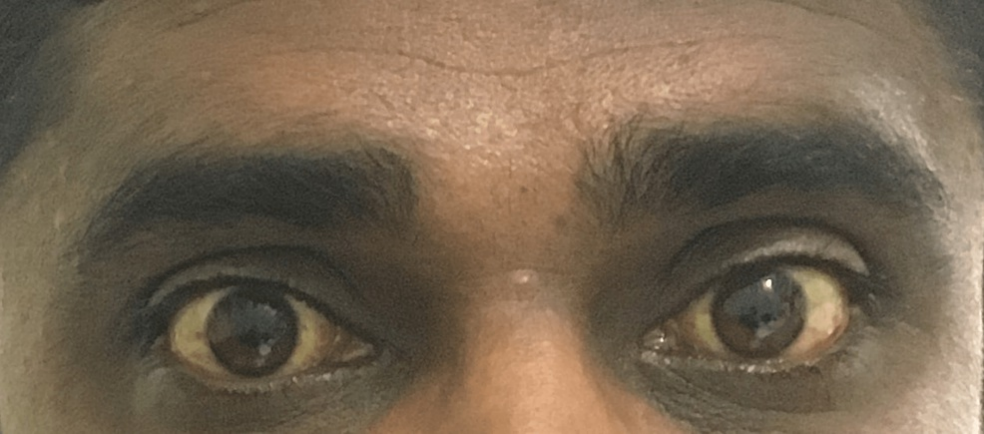
Gradual disappearance of the icterus in six months

A clinic-level reassessment was done after one month. Clinically, he improved, and the discoloration in his eyes subsided (Figure 4).

His liver functions, including bilirubin, improved well. He tapered off the oral steroids. Further exposure to the herbicide MCPA was avoided by the patient as advised. After six months of admission, he made a full recovery without any relapses.

## Discussion

Chlorophenoxy herbicides are used widely for the control of broad-leaved weeds. MCPA is one of the major varieties of chlorophenoxy herbicides, and it is the most common cause of chlorophenoxy poisoning in the North Central Province of Sri Lanka [1]. There is limited information on clinical toxicity from MCPA poisoning, with only six single case reports in the literature [1]. The toxicity occurs through acute oral, dermal, and inhalation methods as well as chronic slow exposure.

MCPA exhibits a variety of mechanisms of toxicity, including dose-dependent cell membrane damage, uncoupling of oxidative phosphorylation, and disruption of acetyl coenzyme A metabolism [2]. Direct cytotoxicity results from the destruction of plasma membranes. The liver is extremely exposed to the hazardous effects of these herbicides because of their vital role in the metabolism of xenobiotics [3]. In vitro studies have demonstrated that MCPA causes dose-dependent hepatotoxicity. Although it is unknown whether lipid peroxidation is a primary or secondary process in cellular toxicity, the depletion of hepatic protective substances including glutathione and protein thiols has also been reported in vitro [4].

Acute and long-term effects of MCPA intoxication have been discussed in terms of toxicology. MCPA can be reproductive, teratogenic, mutagenic, carcinogenic, and poisonous to organs. The kidneys, liver, spleen, and thymus are among the target organs for organ toxicity that have been discovered in animal research. Vomiting and diarrhea, hypotension and bradycardia, acute renal failure, respiratory arrest, reversible anemia, thrombocytopenia, hemolytic anemia, hypocalcemia, gastrointestinal bleeding, liver impairment, and muscle weakness are some of the reported uncommon presentations [2].

In this case, our patient sprayed his plants with MCPA a few months before his presentation. However, he did not follow the requirements listed in the herbicide use guideline and had poor protection when handling the herbicide.

In addition to recently starting spraying herbicide, in a particular instance, our patient had been exposed to a large amount of the same chemical directly as well. He explicitly stated that as he was mixing up the chemical, a lot of mist blew out and sprayed onto his body, including his face. Even if his explanation characterized the considerable exposure to the toxin as acute, it was not possible to measure the amount of the chemical.

After ruling out all other potential causes of the acute hepatic injury, we became suspicious of drug-induced liver injury (DILI) due to the patient’s recent and ongoing exposure to the chlorophenoxy herbicide. The clinical and biochemical findings mainly pointed to the probability of mixed hepatocellular and cholestatic liver damage in DILI. Also, according to the American Academy of Gastroenterology’s clinical recommendations for drug-induced liver impairment, our patient’s R-value fell within the range of persistent mixed pattern levels that suggest DILI [5].

The diagnosis of DILI was confirmed by the histopathologic findings of the liver biopsy, particularly the portal inflammatory infiltration by neutrophils and eosinophils and the cholestasis picture [5].

Toxin-mediated liver damage is caused by a variety of underlying mechanisms, some of which are already known and may apply to our case. Disruption of calcium homeostasis resulting in cell lysis and surface blabbing, canalicular damage, chemical bioactivation by cytochrome P450 to produce reactive species, apoptosis stimulation, mitochondrial damage, and autoimmune stimulation are a few of the underlying processes [6].

The bile canaliculi were damaged because of the chemicals, which ultimately resulted in cholestasis. Also, because of the excess release of bile salts following inflammation, further injury to cell membranes and biliary epithelium occurred, worsening the clinical picture.

## Conclusions

MCPA is one of the widely used herbicides in the Sri Lankan agricultural society. The clinical manifestations that have been documented in the literature as a result of acute or chronic exposure to MCPA herbicide are limited. Liver impairment is also a rare presentation, even though it has not been well studied in the past. Herbicide exposure should be considered in routine clinical practice as one of the key etiologies in patients who come with liver damage and agricultural background.
